# Motivation to change, coping, and self-esteem in adolescent anorexia nervosa: a validation study of the Anorexia Nervosa Stages of Change Questionnaire (ANSOCQ)

**DOI:** 10.1186/s40337-016-0125-z

**Published:** 2017-04-17

**Authors:** Dagmar Pauli, Marcel Aebi, Christa Winkler Metzke, Hans-Christoph Steinhausen

**Affiliations:** 1 Department of Child and Adolescent Psychiatry and Psychotherapy, University Hospital of Psychiatry Zurich, Neumünsterallee 3, 8032 Zurich, Switzerland; 2 Child and Youth Forensic Psychiatry, Department of Forensic Psychiatry, University Hospital of Psychiatry Zurich, Zurich, Switzerland; 3 Clinical Psychology for Children/Adolescents and Couples/Families, Department of Psychology, University of Zurich, Zurich, Switzerland; 4 Clinical Psychology and Epidemiology, Institute of Psychology, University of Basel, Basel, Switzerland; 5 Centre for Child and Adolescent Mental Health, Capital Region Psychiatry, Copenhagen, Denmark

**Keywords:** ANSOCQ, Anorexia nervosa, Adolescents, Motivation to change, Readiness to change, Self- esteem, Coping style

## Abstract

**Background:**

Understanding motivation to change is a key issue in both the assessment and the treatment of eating disorders. Therefore, sound instruments assessing this construct are of great help to clinicians. Accordingly, the present study analysed the psychometric properties of the Anorexia Nervosa Stages of Change Questionnaire (ANSOCQ), including its relation to coping style and self-esteem.

**Methods:**

*N* = 92 adolescents referred to an eating disorders outpatient clinic meeting criteria for anorexia nervosa gave written informed consent to participate in this study and completed the ANSOCQ, the Eating Disorder Inventory, the Eating Attitudes Test, the Body Image Questionnaire, two questionnaires measuring Self-Related Cognitions and the Coping Across Situations Questionnaire. After a treatment period of nine months, clinical anorexia nervosa diagnosis and the body mass index were re-assessed. In addition to exploratory factor analysis, correlational analysis was used to test for the convergent validity of the ANSOCQ and logistic regression analysis was used to test its predictive validity.

**Results:**

The ANSOCQ had good psychometric properties. Factor analysis yielded two meaningful factors labelled as ‘weight gain and control’ and ‘attitudes and feelings’. Internal consistencies of the two factors amounted to Cronbach’s alpha = .87 and .76, respectively. Significant correlations with other scales measuring eating disorder psychopathology were indicative of meaningful construct validity. Higher motivation to change was related to higher self-esteem and a more active coping style. Higher (positive) ANSOCQ total scores predicted remission of anorexia nervosa after nine months of treatment. A higher score on ‘attitudes and feelings’ was a protective factor against drop-out from intervention.

**Conclusions:**

The ANSOCQ is a clinically useful instrument for measuring motivation to change in adolescents with AN. Two factorial dimensions explain most of the variation. Self-esteem and coping style are relevant additional constructs for the understanding of the motivation to change in anorexia nervosa.

**Trial registration:**

NCT02828956. Retrospectively registered July 2016

**Electronic supplementary material:**

The online version of this article (doi:10.1186/s40337-016-0125-z) contains supplementary material, which is available to authorized users.

## Plain english summary

We analysed the utility and the quality of the Anorexia Nervosa Stages of Change Questionnaire (ANSOCQ) to measure motivation to change in patients with anorexia nervosa, and its relation to self-esteem and coping behaviour.

The study was based on the responses of 92 adolescent patients with anorexia nervosa to questionnaires measuring symptoms of AN, motivation to change, coping style, and self-esteem at the beginning of their treatment. Nine month later, the clinical diagnosis and the Body Mass Index were re-evaluated. 

Our results show, that the ANSOCQ is a valid and solid instrument to measure motivation to change, and contains two main factors measuring ‘Weight gain and control’ and ‘attitudes and feelings’. Patients with higher motivation to change showed higher levels of self-esteem and had more active coping strategies, were more likely to recover from their eating disorder after nine months, and were less likely to drop out from treatment. We conclude that the ANSOCQ is a useful instrument for measuring motivation to change in adolescents with AN.

## Background

Recently, the concept of motivation to change and its impact on treatment outcome in eating disorders has gained increasing attention [1–7]. From clinical experience, it is well-known that patients with anorexia nervosa (AN) in particular, are often highly ambivalent towards changing their eating disorder behaviour due to positive illness-maintaining factors reinforcing the symptoms [8] and denial of the illness [9, 10].

Various suggestions including specific therapeutic interventions have been identified to increase patients’ level of motivation to engage in treatment and to assist practitioners to form a therapeutic alliance with the patient [11–15]. A randomized controlled trial by Katzman et al. [16] showed that motivation enhancement treatment (MET) was equally successful in outcome as cognitive behaviour therapy (CBT) as a first phase of treatment. Two recent reviews [5, 6] found some support for the impact of motivational interventions on outcome in eating disorders, mainly on binging, but less so on purging behaviours, and no influence on restrictive eating.

It has been shown that a higher level of motivation in patients with eating disorders is predictive of the treatment process and outcome measures such as a higher quality of the therapeutic alliance and less binge eating [17], greater weight gain [18, 19], lower body dissatisfaction, lower drive for thinness [20] and increase in body mass index (BMI). Across three reviews, pre-treatment motivational level was associated with positive change in weight, restrictive eating and binging, but it did not impact purging behaviours [4, 21, 22].

Several attempts have been made to develop instruments for the specific assessment of the decisional balance between pros and cons of the eating disorder [20, 23–26], or for motivation to change in patients with eating disorders in a more general way without addressing specific symptoms [27–29]. The Readiness and Motivation Interview developed by Geller [23] has shown good psychometric properties [22], and a significant association with change in eating disorder symptoms [30, 31] but needs both interviewer training and considerable time for administration. The Attitudes Towards Change in Eating Disorders Scale (ACTA) developed by Fernandez et al. [29] measures readiness for change by relating different items to a corresponding stage of change. Since each stage of change in the patient is rated separately, this questionnaire is difficult to interpret.

The Anorexia Nervosa Stages of Change Questionnaire (ANSOCQ) developed by Rieger et al. [18] in a sample of adolescent and adult inpatients is a 20-item self-report measure that focuses on crucial motivational issues in the process of changing eating disorder behaviours (such as motivation to gain weight, motivation to overcome symptomatic behaviour, or emotional and interpersonal problems). Factor analysis of the questionnaire in a study by Rieger et al. [32] yielded the three factors ‘*weight gain’*, ‘*eating, shape and weight concerns’* and ‘*ego-alien aspects’*.

The predictive validity of the ANSOCQ has been analysed in two studies with Spanish adolescent patients showing that the ANSOCQ predicts the necessity of hospitalization and weight maintenance [33, 34]. Recently, a positive short-term prediction of weight gain by the ANSOCQ was also shown in a German adolescent sample [7]. Wade et al. [3], in their study based on adolescent and adult inpatients, found that five out of six motivational sub-scores but not the ANSOCQ total score, predicted changes over six weeks in eating disorders psychopathology as measured by the EDE. However, change in ANSOCQ total score after two weeks of treatment was predictive of higher level of change in eating pathology after six weeks. The odds of being discharged as improved were 5.3 times greater for individuals with higher ANSOCQ stage of change in another study [1].

In terms of critical evaluation of previous research of motivation to change in patients with eating disorders, a recent systematic review by Hötzel et al. [21] raised the question of whether separate subscales providing different scores for symptom domains are advantageous over a global total score of motivation to change. According to the authors, this might be important in bulimia nervosa, where the motivation to change dietary restriction has been shown to be rather high compared to the motivation to change bingeing behaviour. The question of whether there are such symptom domain-related motivational subscales remains unclear for instruments to assess motivation in anorexia nervosa patients. In addition, Hötzel et al. [15] stated that for decisional balance scales there is a current lack of evidence for predictive value, whereas the predictive value has been shown for instruments based on the transtheoretical stages of change model.

There are several studies assessing the relevance of maladaptive coping strategies in eating disorder patients, however, these investigations were not focused on the question of motivational change. Villa et al. [35] showed that coping styles were even more powerful than eating disorder features in discriminating healthy individuals from eating disorder patients. Furthermore, eating disorder pathology was related to maladaptive problem-solving in inpatients with AN in a study by Swanson et al. [36]. In a longitudinal community-based study, high-risk adolescents with abnormal eating behaviour shared a very similar pattern across three assessment points between mean ages 14 and 20. These patients were characterized by less active coping and lower self-esteem [37].

## Methods

### Aims

The present study aimed to assess the psychometric properties, including the factorial, structure of the German translation of the ANSOCQ in a Swiss-German sample of adolescent patients with eating disorders. The second aim was to investigate the association of motivation to change with the core psychological constructs of coping style and self-esteem in terms of convergent validity. To our knowledge this is the first study to examine the link between these constructs with motivational issues in anorexia nervosa treatment. We hypothesised that low coping capacity as well as low self-esteem could be linked to a lower motivation to change eating disorder symptoms. The third aim was to replicate the predictive validity findings of the ANSOCQ in terms of higher motivation correlating with higher treatment satisfaction, less treatment refusal, higher probability for remission of the anorexia nervosa diagnosis and increase of the BMI. The study also aims to contribute to the discussion of whether motivation to change in anorexia nervosa patients is an overall general readiness to change, or should rather be regarded as a multifactorial construct.

### Sample and procedure

Out of 99 adolescents consecutively referred to our specialist eating disorders clinic of the Department of Child and Adolescent Psychiatry, University of Zurich meeting criteria for AN a total of 92 (92.9%) adolescents gave written informed consent to participate in this validation study. Patients who refused to take part in the study had a mean BMI of 16.6 (SD 1.19, range 15.5–18.2). Neither BMI z-scores nor age differed significantly between the 92 participants and the 7 non-participants. The sample of the 92 participants consisted of 87 (94.6%) females and five (5.4%) males aged between 12.0 and 18.6 (Mean = 15.6, SD = 1.4) years.

The mean BMI of the entire patient sample was 16.4 (SD 1.22, range 12.9–18.8). BMI was below the 3^rd^ percentile for age - standardized charts for all patients at the time of diagnosis (except for three patients discharged from a somatic hospital with a previous diagnosis of AN). A clear age-related cut-off point is needed to define thinness in adolescence, considering the fact that BMI in childhood and adolescence changes substantially with age [38]. Therefore, we used the procedure (LMS-method) as described by Cameron [39] and Cole et al. [38, 40]. This method summarises the data in terms of three smooth age specific curves called L (lambda), M (mu), and S (sigma). The assumption underlying the LMS method is that after BoxCox power transformation the data at each age are normally distributed in order to obtain standardized z-scores (age-corrected body mass indices). This procedure allows the definition of thinness in different age-groups and the joint analyses and comparisons of different age-cohorts. The original data on the LMS-parameters and on the percentiles stem from the American National Centre for Health Statistics for Ages 5–19 years, and have been adapted to Switzerland [41]. The scores for age corrected BMI (z-scores) of the sample ranged from -4.13 to–0.18 (mean = -1.78, SD = 0.75).

All patients fulfilled ICD – 10 criteria for AN with 84 (91.3%) having the restricting type and 8 (8.7%) the purging type of the disorder. The diagnosis was based on best evidence practice with the final diagnosis being made by two experienced senior clinicians in multidisciplinary teams. The duration of illness (DUI) differed between participants with four out of five patients being newly diagnosed with AN (79.7%). A high proportion of patients had a short DUI prior to admission (mean = 1.02, SD = 1.09, range 0.1–6.2 years). The mean was inflated by five outliers with DUIs between 2.5 and 6.2 years. Treatment involved weekly individual psychotherapeutic sessions with the patients, as well as family-based therapy. Four patients were treated in the inpatient unit (4.3%) and 88 in the outpatient unit (95.7%).

### Measures

#### Anorexia Nervosa Stages of Change Questionnaire (ANSOCQ)

The ANSOCQ is a 20-item self-report questionnaire designed to evaluate readiness to change in patients with AN [42]. It includes items addressing eating behaviour, aspects of weight and body shape, methods of weight control, as well as related emotional and relationship problems. The ANSOCQ is based on the trans-theoretical concept of motivational stages, which has been developed by Prochaska & Di Clemente [43]. Each item refers to a specific symptom and participants can choose between five answers representing the five stages of change. Corresponding to this model, and based on the total item score (possible range 20–100), patients can be classified into the five different stages of motivation, i.e., *pre-contemplation* (score 20–29), *contemplation* (score 30–49), *preparation* (score 50–69), *action* (score 70–89), and *maintenance* (score 90–100). A low total score means low motivation to change the eating disorder. The German translation of the questionnaire was performed by the authors of the present study with kind permission by the authors of the original version.

In previous studies, the ANSOCQ showed good internal consistency and one-week test-retest reliability both in the English and Spanish version [18, 33]. Good construct-related validity was demonstrated by significant correlations between ANSOCQ scores and other constructs of readiness to change, decision balance and self-efficacy [42]. The ANSOCQ also demonstrated predictive validity insofar as low scores correlated with the necessity of hospital admission [34], and high scores with weight maintenance after inpatient treatment [44].

#### Eating Disorder Inventory (EDI-2)

The EDI-2 is a 91 item self-report measure assessing a broad variety of symptoms and attitudes related to eating disorders. It consists of 11 subscales (‘*drive for thinness’, ‘bulimia’, ‘body dissatisfaction’, ‘effectiveness’, ‘interpersonal distrust’, ‘interoceptive awareness’, ‘asceticism’, ‘perfectionism’, ‘social insecurity’, ‘maturity fears’ and ‘impulse regulation’*). The EDI-2 shows good psychometric properties in both the original and the German version [45, 46].

#### Eating Attitudes Test (EAT)

The EAT is a 40 item self-report measure assessing eating attitudes and symptoms related to eating disorders. In consists of the 3 subscales ‘*dieting’, ‘bulimia’ and ‘oral control’* [47]. The German translation by Steinhausen [48] was used in the present study.

#### Body Image Questionnaire (BIQ)

The Body Image Questionnaire is a self-report measure assessing different aspects of body perception based on the model of semantic differentials. The questionnaire requests a self-description of the body in terms of 16 bipolar adjectives each (e.g. “beautiful-ugly”) [49]. The German version of the questionnaire [48] has been subjected to factor analysis resulting in two subscales measuring ‘*unattractiveness’* and ‘*body-mass’* (in terms of massiveness or bulk) both in adolescent patients with eating disorders and controls and α - coefficients of .93 and .81, respectively [50].

#### Self-related Cognitions Questionnaire (SRCQ)

This questionnaire contains two scales. Self-esteem was measured by the total score of the classical ten-item scale by Rosenberg [51], and self-awareness in terms of a total score by a German questionnaire designed by Filipp and Freudenberg [52]. The latter scale assesses introspective capacities for one's feelings, actions, and past (e.g. “I quickly notice mood swings”). The two scales had α - coefficients ranging from 0.77 to 0.89 across three assessments in a large community-based study in adolescents [53].

#### Coping Across Situations Questionnaire (CASQ)

Our modified version of the German Coping Across Situations Questionnaire [53] addresses coping in four problem areas with school, parents, peers, and the opposite sex. Factor analysis of the 20 items resulted in two scales measuring active coping represented by items like “I discuss problems with my parents or other adults”) and avoidant behaviour (represented by items like “I try to forget my problems by taking alcohol or other drugs”) and α - coefficients of internal consistency for the two scales ranged from .65 to .70 in various age and sex strata of a community sample of 11–17 year old adolescents [54].

#### Treatment outcome

Three variables were defined for measuring treatment outcome, namely, treatment satisfaction as rated by the treating clinician, drop-out from treatment, and remission of AN (not fulfilling ICD-10 criteria anymore).

### Statistical analyses

Factor extraction of exploratory factor analysis (EFA) was conducted using robust maximum likelihood estimates with Varimax rotation of the resulting factors. For determining the number of factors, a parallel analysis (a Monte Carlo simulation technique with 500 samples, criterion 95th percentile) was performed. A factor was retained if the associated eigenvalue was larger than the 95th percentile of the distribution of eigenvalues derived from the random data. Parallel analyses were found to be robust, appropriate, and superior compared to other methods (e.g., Kaiser criteria, Cattell's Scree test) to determine the number of factors.

In addition, a confirmatory factor analysis (based on robust maximum likelihood estimates) was performed allowing latent factors to be correlated and allowing items to load on both factors simultaneously. For estimation the goodness of fit, the root mean square error of approximation (RMSEA) including a parsimony correction and the comparative fit index (CFI) for evaluating the hypothesized model compared to a null model was taken into account. Acceptance of any model was based on the following cut-offs: RMSEA < .06 and CFI > .95 [55]. Furthermore, internal consistency coefficients (Cronbach̕s alpha [56]) were calculated for the resulting scales and various correlation coefficients were computed. Finally, linear and logistic regressions were used to analyse the predictive validity of the ANSOCQ regarding several measures of treatment outcomes. Analyses were conducted in Mplus 7.31 [57] for Mac OS X.

## Results

This study assessed the psychometric properties of the ANSOCQ, including the factorial structure, in a Swiss-German sample of adolescent patients with eating disorders. Furthermore, the study investigated the association of motivation to change with the core psychological constructs of coping style and self-esteem in terms of convergent validity. Lastly, it assessed its predictive validity in terms of treatment outcome.

Descriptive findings of the sample before and after treatment are shown in Table 1. After a treatment period of nine months, BMI outcome data of 86 patients were available. At this time, the age range of the patients was between 12.8–19.9 years (mean = 16.5 years, SD = 1.36) and the BMI of the sample ranged from 13.2 to 24.4 (mean = 18.49, SD = 1.81). The BMI was significantly improved (*t* = 10.16, *df* = 85, *p* < 0.001). According to the ratings made by the treating clinicians, treatment satisfaction was good or very good in 78.2% of the sample. At follow-up, out of the 88 diagnosed patients, 58 (65.9%) patients no longer fulfilled the diagnostic criteria of AN and of the remaining 30 (34.1%) patients of the sample 20 (22.7%) patients fulfilled the diagnostic criteria of AN restrictive type, 5 (5.7%) patients of AN binge-eating/purging type and 5 (5.7%) patients were diagnosed with an atypical AN.

**Table 1:** Descriptive findings of the sample before and after treatment (*N* = 92)

	Mean (SD)/n (%)
Female sex (n)	87 (94.6%)
Age (years) Pre-treatment measures:	15.64 (1.40)
Illness duration before treatment (years)	1.05 (1.10)
Inpatient treatment (n)	4 (4.3%)
AN restrictive subtype (n)	84 (91.3%)
AN purgative subtype (n)	8 (8.7%)
Weight before treatment (kg)	44.46 (4.89)
BMI before treatment (kg/m^2^)	16.42 (0.75)
BMI (z-score) before treatment	−1.78 (0.75)
Post-treatment measures:	
Treatment satisfaction good or very good (n)^1^	61 (78.2%)
Treatment refusal (n)^2^	11 (12.5%)
Remission of AN (n)^2^	58 (65.9%)
BMI after treatment (kg/m^2^)^3^	18.50 (1.81)
BMI change after treatment (kg/m^2^)^3^	2.01 (1.84)

*AN* anorexia nervosa, ^1^missings = 14, ^2^missings = 4, ^3^missings = 6

On the five different stages of motivation of the ANSOCQ 12 (13.0%) patients were in the *pre-contemplation stage*, 45 (48.9%) patients in the *contemplation stage*, 32 (34.8%) in the *preparation stage*, 3 (3.3%) patients in the action stage, and no patient in the *maintenance stage* of change. The ANSOCQ total score ranged from 23 to 72 (mean = 47.5, SD = 12.8).

Factor analysis based on robust maximum likelihood estimates revealed two factors with Eigenvalues greater than the 95th percentile of the Eigenvalues of the parallel analyses (based on 500 random samples): A first factor with an Eigenvalue of 6.78 (95th percentile of the Eigenvalues from parallel analyses = 2.19) and a second factor with an Eigenvalue of 2.08 (95th percentile of the Eigenvalues from parallel analyses = 1.93). Compared to the one-factor and three-factor solution, the two-factor solution was also most appropriate according to the CFI (two-factor CFI = 0.85, one-factor CFI = 0.78, three-factor CFI = 0.82) and the RMSEA (two-factor RMSEA = 0.073, one-factor RMSEA = 0.082, three factor RMSEA = 0.085). Thus we decided to use the two factor solution in further analyses.

Out of the 20 ANSOCQ items, nine items loaded uniquely on the first factor and seven items loaded uniquely on the second factor, with one item (#12) loading on both factors with *p* < .05. Three items did not show significant factor loadings. Omitting these three items resulted in an improved model with acceptable fit indices (RMSEA = 0.046, CFI = 0.95). The subsequent confirmatory factor analysis used robust maximum likelihood estimates based on the previously defined two factor solution from EFA but allowed correlated factors and item 12 to load on both factors simultaneously. The final two factor model showed a further improved fit to the data (CFI = 0.95, RMSEA = 0.041).

This final two-factor solution is presented in Table 2 and contains two psychologically meaningful factors based on a well-balanced item-pool of ten and eight items. The items with the highest loadings on factor 1 are directly related to weight gain in general (item 1 and 8) or weight gain related aspects (e.g. weight gain of certain body parts in item 3, change of the relation of fat versus muscles in item 9, rate of accepted weight gain in item 10). Some items also refer to food intake (e.g. frequency of meals in item 13, avoided foods in item 12). Thus, factor 1 was labelled ‘*weight gain and control’*. The internal consistency of the factor 1 sub-scale amounted to α = .87. The items with the highest loadings on factor 2 refer to eating disorder related cognitions and feelings (e.g. preoccupations with food or weight in item 14, importance of body shape for personal happiness in item 6, fear of becoming fat in item 7), general emotional problems, or personality characteristics (emotional problems in item 18, and personality in item 19). Consequently, factor 2 was named ‘*attitudes and feelings’*. Internal consistency of the resulting sub-scale amounted to α = .76. The two factors of the ANSOCQ were moderately correlated (*r* = 0.58; *p* < .01). It should be noted that higher scores on these two scales imply less pathology in terms of a higher motivational level to overcome the eating disorder.

**Table 2:** Factor loadings based on robust maximum likelihood estimates with varimax-rotation of the 20 ANSOCQ items

	Item	Factor 1 weight gain and control	Factor 2 attitudes and feelings	Factor- number according to Rieger and Touyz (2006)^a^
3	Weight gain: body parts	**0.79** ^a^	−0.05	1
1	Weight gain	**0.76** ^a^	0.06	1
5	Weight gain and health	**0.67** ^a^	0.17	1
9	Weight gain: fat versus muscle	**0.66** ^a^	0.21	1
8	Weight loss	**0.61** ^a^	0.16	3
10	Rate weight gain	**0.58** ^a^	−0.04	1
13	Daily eating	**0.55** ^a^	0.24	3
4	Weight and appearance	**0.50** ^a^	0.08	1
2	Normal weight	**0.49** ^a^	0.08	1
12	Avoided foods (fat)	**0.40** ^a^	**0.40** ^a^	3
14	Preoccupations	−0.10	**0.80** ^a^	2
19	Personality	−0.13	**0.58** ^a^	3
7	Fear of fat	−0.12	**0.57** ^a^	2
6	Body importance	0.19	**0.54** ^a^	2
18	Emotional problems	−0.02	**0.49** ^a^	3
17	Weight control methods	0.24	**0.49** ^a^	2
11	Body standards	0.22	**0.43** ^a^	2
16	Eating feelings	0.37	0.43	2
15	Eating behaviour	0.30	0.12	2
20	Interpersonal problems	−0.06	0.32	3
	Eigenvalue	6.78	2.08	

Factor loadings of the final scales are marked in bold. ^a^Factor names according to Rieger and Touyz [32]: 1 = Weight Gain Factor, 2 = Eating, Shape and Weight Concerns, 3 = Ego-Alien Aspects

Various correlations were computed to test for convergent validity of the two ANSOCQ factor sub-scales. As Table 3 shows, there were significant negative correlations between both BMI and age adjusted BMI (based on percentile charts), respectively, and the stage of change classification by the ANSOCQ in this sample, indicating that a lower BMI was associated with a higher motivation to change. Furthermore, the ANSOCQ subscale 1 (‘*weight gain and control’*) showed highly significant negative correlations with the EDI-2 scales measuring ‘*drive for thinness’* and ‘*body dissatisfaction’,* and moderately significant negative correlations with the EDI-2 scales measuring ‘*bulimia’* and ‘*asceticism’*. Thus, the motivation to overcome weight gain and control increased with decreasing eating disorder pathology as measured by these scales. ANSOCQ factor 2 subscale (‘*attitudes and feelings’*) had only a single and moderate negative correlation with the EDI-2 scale measuring ‘*body dissatisfaction’*. Again, higher scores in the motivational domain were associated with less body dissatisfaction.

**Table 3:** Correlations between the ANSOCQ scales and further eating disorder characteristics

	Mean (SD)	ANSOCQ scale 1	ANSOCQ scale 2	ANSOCQ total score
BMI	16.42 (0.75)	−.29**	−.28**	−.33**
BMI (age corrected z-score)	−1.78 (0.75)	−.29**	−.36***	−.37***
Eating Disorder Inventory (EDI-2)				
Drive for thinness	26.97 (9.86)	−.61***	−.23*	−41***
Bulimia	13.00 (6.39)	−.19	−.14	−.17
Body dissatisfaction	33.14 (10.58)	−.57***	−.23*	−.47***
Ineffectiveness	26.91 (7.97)	−.30**	−.13	−.23*
Perfectionism	18.89 (6.26)	−.11	.06	−.02
Interpersonal distrust	19.00 (5.63)	−.28**	−.14	−.25*
Interoceptive awareness	30.72 (9.51)	−.15	.02	−.06
Maturity fears	24.96 (6.66)	−.12	.04	−.04
Asceticism	20.12 (6.28)	−.28**	.04	−.17
Impulse regulation	25.16 (7.47)	−.13	.01	−.06
Social insecurity	22.79 (6.62)	−.20	−.17	−.20
Eating Attitudes (EAT)				
Dieting	14.34 (10.20)	−.56***	−.31*	−.49***
Bulimia	5.03 (3.53)	−.24*	−.04	−.15
Oral control	9.60 (4.79)	−.22*	−.01	−.14
Body Image Questionnaire (BIQ)				
Attractiveness	26.60 (8.60)	.09	.04	.07
Body-Mass	21.77 (6.79)	−.52***	−.25*	−.48***

* = significance (two sided), *p* < .05, ** = significance (two sided), *p* < .01, *** = significance (two sided), *p* < .001

In addition, there was a highly significant negative correlation between ANSOCQ factor 1 subscale (‘*weight gain and control’*) and the EAT subscale measuring ‘*dieting’,* whereas ANSOCQ factor 2 sub-scale (‘*attitudes and feelings’*) was moderately and negatively correlated with this subscale of the EAT. The two factor-based ANSOCQ subscales had significant negative correlations with the subscale measuring perceived ‘*body-mass’* of the BIQ. Finally, ANSOCQ total score also showed highly significant negative correlations with the EDI-2 scales ‘*drive for thinness* and *dieting’,* the EAT subscale ‘*dieting’* and the subscale *body mass* of the BIQ. The negative correlations indicate that the motivation to change as measured by the two factor-based subscales increased with decreasing eating disorder pathology.

Table 4 presents further correlation coefficients of the two ANSOCQ scales with selected personality constructs. Both the ANSOCQ factor 1 subscale (‘*weight gain and control’*) and the ANSOCQ total score correlated positively with self-esteem, whereas the ANSOCQ factor 2 subscale (‘*attitudes and feelings’*) showed a significant positive correlation with self-awareness. Furthermore, both factors were positively associated with active coping. No significant correlation between age and ANSOCQ factor 1 subscale (‘*weight gain and control’*; *r* = -.02, *p* >0.05) could be found. However, older patients showed a higher motivational stage as measured by the ANSOCQ factor 2 sub-scale (‘*attitudes and feelings’*; *r* = .24, *p* <0.05).

**Table 4:** Correlations between the ANSOCQ scales and other psychosocial measures

	Mean (SD)	ANSOCQ scale 1	ANSOCQ scale 2	ANSOCQ total score
Self-related cognitions scales (SRCQ)				
Self-esteem	19.64 (17.36)	.35**	.16	.30**
Self-awareness	21.63 (6.20)	−.08	.15	.05
Coping strategies scales (CASQ)				
Active coping	19.76 (4.77)	.23*	.31**	.31**
Avoidant coping	11.91 (5.12)	−.06	−.17	−.13

* = significance (two sided), *p* < .05, ** = significance (two sided), *p* < .01, *** = significance (two sided), *p* < .001

Neither the ANSOCQ total score (β = 0.17, *t* = 1.54, *p* > 0.05), nor the ANSOCQ factor 1 subscale (*weight gain and control*; β = 0.04, *t* = 0.27, *p* > 0.05) or the ANSOCQ factor 2 subscale (*attitudes and feelings*; β = 0.17, *t* = 1.16, *p* > 0.05) predicted BMI increase in multivariate linear regression analyses with the ANSOCQ scores as predictors and age as covariate. Results of the logistic regression analyses for predicting treatment satisfaction, treatment drop – out, and remission of AN are presented in Table 5. High ANSOCQ total scores were predictive of remission from AN. In addition, a high ANSOCQ factor 2 sub-scale score measuring ‘*attitudes and feelings’* was protective against drop - out from treatment. Further multivariate logistic regression analyses of the total, as well as the two factor sub-scores of the ANSOCQ and the eating attitudes scale total scores as predictors for remission and drop-out from treatment, showed no significant correlations. We did not perform any multivariate statistics with the subscales of the EDI-2 to avoid type-1 and type 2 errors due to the limited sample size.

**Table 5:** Prediction of treatment outcome

Outcomes	Treatment satisfaction OR (95% CI)	Treatment refusal OR (95% CI)	Remission of AN OR (95% CI)
ANSOCQ total score	1.01 (0.97–1.06)	0.97 (0.92–1.02)	1.04 (1.01–1.08)*
ANSOCQ scale 1	0.98 (0.91–1.06)	1.04 (0.96–1.13)	1.03 (0.96–1.10)
ANSOCQ scale 2	1.07 (0.94–1.22)	0.84 (0.71–0.99)*	1.08 (0.96–1.22)

*CI* confidence interval, *OR* odds ratios

Age was included as covariate in all analyses

* = significance (two sided), *p* < .05

## Discussion

In general, the present results confirm previous studies reporting good psychometric properties of the ANSOCQ [33, 42]. The mean age of our Swiss sample was younger than in the Australian original studies [18, 32, 42] and similar to the Spanish studies [33, 34] and a recent German study [7]. The interpretation of our results has to consider the special conditions of our treatment unit. The patients were frequently recruited within the first phase of their illness while experiencing their first weight loss, and almost of all of them (95.7%) were initially treated as outpatients.

As most of these young patients were not voluntarily admitted for treatment in the early stages of the illness, these patients with an average duration of illness of 1.02 years presented with rather low motivation to change at the time of referral. Compared to the older original sample by Rieger et al. [42], our younger patients clearly had lower levels of motivation with only 3.3% of patients in the action and none in the maintenance state, compared to 20.5% in action or maintenance state in the sample by Rieger et al. [42].

Our factor analysis provided a clinically meaningful solution, with a clear distinction of only two factors, namely, factor 1 (‘*weight gain and control’*) and factor 2 (‘*attitudes and feelings’*). All factor 1 items are clearly related to aversion to gaining weight (items 1, 2, 4, 5, 10 in general or items 3, 9 in certain body-parts), and the desire to lose weight (item 8) or behaviours directly connected to gaining or losing weight (item 13). The items loading on factor 2 refer to cognitions or emotions in association with eating disorder symptoms (items 6, 11 and items 7, 14, 17) or general personal or emotional problems (items 18, 19). Item 12 (avoiding certain foods like fat), which loads on both factors, refers to cognitions with high anxiety level but also to behaviours directly related to gaining weight. Consequently, this item contributed to both scales. The two sub-scales both showed good internal consistencies so that these factor-based sub-scales are useful for research and clinical practice.

The modest intercorrelation of the two ANSOCQ factors in our study are not completely in line with the analysis by Rieger & Touyz [32] who found that their ANSOCQ factors were highly intercorrelated, leading to the assumption that there is an underlying general factor of readiness to change in patients with AN. In younger patients the motivation to gain weight might be crucial, and the motivation to deal with emotional aspects might not yet be sufficiently developed in the early stage of the illness. This assumption is corroborated by our finding of higher motivation as measured by factor 2 sub-scale of the ANSOCQ with increasing age. In general, Factor 1 has more and higher essential loadings, a higher Eigenvalue, and explains more of the total variance than Factor 2. This indicates that the general motivation in our young patients seems to be based mostly on their level of motivation to gain weight. The lower item loadings on the ‘*Ego-Alien aspects*’ scale by Rieger & Touyz [32] may be due to the rather young age of our patients and their early stage of the eating disorder. This finding corresponds to the clinical experience that younger patients with an early stage of an eating disorder are less aware of negative consequences of the illness, and therefore experience most of the symptoms as rather ego-syntonic or may ignore ego-dystonic aspects.

Furthermore, we found a negative correlation between ANSOCQ scores and BMI, which is inconsistent with the results of the study by Serrano et al. [33]. Our findings are in line with two studies [7] indicating that the BMI at admission was inversely correlated to motivation to change. One of theses studies [1] showed that more severe eating disorder pathology as measured by the EDI-2 was associated with lower motivation to change. It may be that young patients within the first phase of weight-loss, who are not yet confronted with the disadvantages of their illness, are determined to lose more weight and have less motivation to change their attitudes and behaviour. Thus, in the early phase of intervention, a lower weight might lead to a higher level of insight into the disadvantages of the eating disorder.

Various additional findings of the present study point to convergent validity of the two newly identified ANSOCQ factor-based subscales, indicating less eating disorder pathology with increasing motivation to change. The negative correlations of the two ANSOCQ sub-scores with the EDI-2 scales *‘drive for thinness’* and *‘body dissatisfaction’* as well as with the EAT subscale *‘dieting’* and the BIQ subscale of perceived *‘body mass’* indicate the high relevance of concerns about body shape and weight representing a basic obstacle for young patients with AN to motivate themselves to overcome their disease. These high correlations confirm the results by Serrano et al. [33] and Rieger et al. [18]. We also found more moderate correlations with the EDI-2 scales measuring ‘*ineffectiveness’, ‘interpersonal distrust’ and ‘asceticism*’. However, the latter studies found that even more EDI-2 subscales (‘*interoceptive awareness’, ‘social insecurity’, ‘maturity fears’*) were correlated with ANSOCQ scores than the present study. Again, it may well be that in the very early stage of illness the motivation of very young patients is mainly focussed on body and weight related issues, whereas other aspects might only become apparent in the later course of the illness. It may well also be the case that these subscales of the EDI-2 are perhaps not as salient for current participants due to overall maturity level.

Consistent with this assumption, we found a high correlation between the ANSOCQ factor 1 sub- scale and the EAT sub-scale 1 (‘*dieting’*) and only a moderate correlation for EAT sub-scale 2 (‘*bulimia’*) and 3 (‘*oral control’*). Again, dieting seems to overshadow other aspects that might account for more motivational variance in a later state of the illness. Further evidence pointing to convergent validity of the two newly identified ANSOCQ sub-scales comes from the significant correlation between perceived high body-mass, as measured by the BIQ, and low motivation to change. Clinical observation indicates that young patients with anorexia nervosa frequently report strong feelings of plumpness, and they often consider the circumference of certain body parts as their greatest problem. Our findings provide support for the conceptualization of an underlying general motivational factor basic to the different cognitive and emotional aspects of the construct of motivation in young patients with eating disorders. For this subgroup of patients in an early stage of their illness, the most useful indicator might be the change in the total motivation score assessed during the first weeks of treatment. In contrast, in older adolescents and adult patients the assessment of various aspects of motivation referring to specific symptoms or symptom clusters may become more important.

In terms of convergent validity, motivation to change in our study was also related to domains other than those assessing core eating disorder symptoms. In a previous study, it has been shown that patients with eating disorders in general have lower self-esteem [58]. Whereas in this study motivation to change was not assessed, the present study found high self-esteem related to higher motivation to change (ANSOCQ factor 1 and ANSOCQ total score). Symptoms of AN tend to be very ego-syntonic and patients often stabilize their self-esteem by being thin. Thus, patients with low self-esteem might show less motivation to give up symptoms that help them to gain self-confidence. In a sample containing older patients with generally more introspective capacities this relation might even be stronger.

Furthermore, we studied the association of coping capacities with the ANSOCQ total score, and subscale scores. We examined the relation of these eating disorder related constructs specifically with motivation to change. There was a positive correlation between active coping strategies and motivation to change mainly for ANSOCQ factor 2 subscale. Therefore, maladaptive coping strategies are not only predictive of eating disorders as suggested by Villa et al. [35], but also of a lower motivation to overcome the disease. In addition, the positive correlations of self-esteem with the ANSOCQ factor 1 subscale and total score are noteworthy, and provide an extension of the finding that low self-esteem is a relevant predictor of stability of abnormal eating behaviour in adolescence [37]. Higher self-esteem and a more active coping style may be connected to higher self-effectiveness, so that the patients with higher self-esteem and more active coping strategies show more motivation to change because they feel more capable of initiating a change.

The last section of the analyses dealing with predictive validity demonstrated the clinical usefulness of the ANSOCQ in terms of predicting the probability of remission and drop-out, but not of BMI increase at 9 month follow up. Previous studies on this issue have revealed contradictory findings. The initial study by Rieger et al. [18] showed a predictive value of the ANSOCQ for BMI increase within the following week after ANSOCQ application, but could not find a prediction of BMI increase after 9 weeks According to the authors this was due to the small sample size. Wade et al. [3] did not find that the total ANSOCQ score at admission was predictive of BMI increase, but the ANSOCQ score change within the first two weeks of treatment was. In a recent German study short-term weight gain was predicted by ANSOCQ scores at admission [7]. Both studies [3, 7] examined adolescents in inpatient units and short-term outcome only whereas our sample was mainly treated in an outpatient unit and outcome was measured after 9 months. In the Spanish study of an adolescent sample by Amettler et al. [34] the ANSOCQ was not applied at admission, but at different points of treatment, and was predictive of the need to hospitalize but not of BMI increase. In sum, it might well be that the predictive value of the ANSOCQ differs in relation to the time of assessment and modality of treatment, and that the change of the level of motivation within the first weeks or months of the treatment is even more predictive than the measurement of motivation at one single point of the disorder.

There are limitations of the present study. First, assessments were not performed by an independent researcher, but by the treating clinicians or based on self-rating questionnaires. In addition, clinical AN diagnoses were not based on a structured clinical interview, but made by two expert clinicians.

## Conclusions

The present data support the psychometric properties of the ANSOCQ in terms of various aspects of validity and internal consistency. Future studies could also assess the long-term stability or motivational changes across time by completing ANSOCQ at baseline and after treatment. Although in clinical practice the ANSOCQ may prove a useful tool for young patients to measure motivation to change, its long-term predictive validity may be less useful for young patients in an early stage of illness. Future research should also clarify whether change of motivational level during early treatment might be a more helpful means to predict outcome than a single measurement at the start of treatment. The identified two factors of the ANSOCQ may serve to create a shorter and less complicated ANSOCQ version. However, the latter would need confirmatory studies. As young patients with AN present themselves with rather low motivation to change in the first phase of the illness, there is a need to assess motivational aspects more systematically to enhance therapeutic strategies, in particular in this specific age group. The consideration of the constructs of coping strategies and self-esteem might be helpful to enhance motivational strategies for young patients with eating disorders.

## Additional file

Additional file 1: Dataset used for the analysis in this study. (XLSX 30 kb)

## Abbreviations

ACTA: The attitudes towards change in eating disorders scale
AN: Anorexia nervosa
ANSOCQ: Anorexia nervosa stages of change questionnaire
BIQ: Body image questionnaire
BMI: The mean body mass index
CASQ: Coping across situations questionnaire
CBT: Cognitive behaviour therapy
CFI: Comparative fit index
DUI: Duration of illness
EAT: Eating attitudes test
EDI: Eating disorder inventory
EFA: Exploratory factor analysis
L: Lambda
M: Mu
MET: Motivation enhancement treatment
RMSEA: Root mean square error of approximation
S: Sigma
SRC: Self-related cognitions
